# Spiking neural networks provide accurate and time-efficient models for whisker stimulus classification of the awake mouse

**DOI:** 10.3389/fnins.2026.1605209

**Published:** 2026-04-01

**Authors:** Steffen Albrecht, Jens R. Vandevelde, Edoardo Vecchi, Gabriele Berra, Davide Bassetti, Maik C. Stüttgen, Heiko J. Luhmann, Illia Horenko

**Affiliations:** 1 Institute of Physiology, University Medical Center of the Johannes Gutenberg University Mainz, Mainz, Germany; 2 Undergraduate Education (FBDTI), Department of Innovative Technologies, University of Applied Sciences and Arts of Southern Switzerland, Lugano-Viganello, Switzerland; 3 Department of Mathematics, Artificial Intelligence in Mathematics, TU Kaiserslautern, Kaiserslautern, Germany; 4 Institute of Pathophysiology, University Medical Center of the Johannes Gutenberg University Mainz, Mainz, Germany

**Keywords:** electrophysiology, local field potential, machine learning, neural prosthesis, neuroprosthetics, spiking neural networks, whisker stimulus classification

## Abstract

Machine learning algorithms have great potential for classifying brain activity, and lightweight classifier algorithms, requiring little computational resources, can be used on low-energy neuromorphic hardware designed for implantable neuroprosthetics. One of these efficient algorithms, the Liquid State Machine, implements the concept of Spiking Neural Networks and has been shown to achieve outstanding results on the task of whisker stimulus detection from the mouse barrel cortex, a widely used model system. While this is promising for neuroprosthetics, it has been unclear how a Spiking Neural Network or other machine learning algorithms perform on data recorded from awake mice and how trained models generalize across individuals, the latter being relevant to transferring trained models to new hardware. Using laminar multi-electrode local field potential recordings obtained from four mice performing a single-whisker detection task, we benchmarked the performance of a collection of lightweight classification algorithms. We found that the Liquid State Machine, a generalized linear model, and the time series classifier ROCKET are the most accurate for stimulus detection. Among those, the Liquid State Machine achieved the fastest model training and inference runtime and provided robust accuracy across individual mice. Additional analyses show that there is no significant improvement in using multiple cortical layers as input for the model and that 40 ms of stimulus recording is sufficient to maintain high detection accuracy.

## Introduction

The development and improvement of brain-machine interfaces (BMI) strongly advanced during the last decades to process brain signals for the control of an external computer or machine (Rapeaux and Constandinou, 2021; Sitaram et al., 2017). BMIs help paralyzed patients suffering from severe brain damage or disrupted connectivity between different brain regions, as typically caused by, for example, spinal cord damage, stroke, or neurodegenerative diseases such as amyotrophic lateral sclerosis (Semprini et al., 2018). BMIs can read out and interpret brain activity to control external actuators such as robotic arms or to function as a spelling interface, thus enabling patients to write text and communicate with their social environment (Rezeika et al., 2018; Sunny et al., 2016).

Instead of turning neural activity into an action performed by a computer or machine, the interface can also create a signal to be sent back to the brain. This would result in a closed-loop system that creates artificial stimulation for the brain based on the signal it receives from it. Such systems are called brain-machine-brain interfaces (BMBIs) and are promising for neural prostheses to replace impaired or even missing biological functionality (Krucoff et al., 2016). For instance, it has been shown that such closed-loop systems can bridge damaged neural pathways in the rat brain after traumatic brain injury by interpreting action potentials captured by multielectrode arrays (MEA) implanted in the cerebral cortex (Guggenmos et al., 2013).

MEAs present several advantages when considered for a neural prosthesis, as the shape of the MEA probe can be tailored to each individual case and is flexible enough to adapt to different brain regions. They provide electrophysiological recordings at a high spatiotemporal resolution that can be further processed differently. One option is to use the local field potential (LFP), i.e., the down-sampled and low-pass filtered raw signal, which includes frequency components below 300 Hz (Petschenig et al., 2022). The LFP reflects the gross spatially weighted average of membrane potential fluctuations of thousands of neurons within a few hundred microns around the electrode (Buzsáki et al., 2012). Another option is to extract action potentials or spikes from the high-frequency signal, usually band-pass filtered between 300 and 3,000 Hz. In the post-processing of the signal (spike sorting), spikes are categorized as single units if the waveform can be clearly identified as action potentials of a single neuron. Otherwise, if spikes are overlapping, they are accumulated as linear combinations of action potentials from small neuron populations in the vicinity of the recording electrode, called multi-unit activity (Rossant et al., 2016). In both cases, computational approaches implemented on the neural prosthesis are challenged to interpret the incoming data accurately. An additional requirement is that these approaches must operate on small-scale and low-powered hardware that is suitable for chronic implantation but provides limited computational resources.

To explore the capability of computational methods to comply with these constraints in a BMBI scenario, the rodent barrel cortex provides an advantageous experimental model (Feldmeyer et al., 2013). Its prominent organization in cortical columns provides a one-to-one topographic representation of single whiskers, sensory organs on the animal’s snout that can be mechanically stimulated in a well-controlled manner (Staiger and Petersen, 2021). Whisker stimulation evokes a localized response in the barrel cortex that can be captured with MEAs and analyzed with the appropriate computational approaches like machine learning (ML) classification algorithms. Based on such recordings, it has been shown that ML classification algorithms, or classifiers, can accurately identify the cortical depth of the recording electrode and the deflection intensity of the whisker stimulus (Wang et al., 2018a; Wang et al., 2018b). While these authors showed that a pre-trained model can be applied for classification on a small microchip within a reasonable amount of time, they did not investigate if it is feasible to train ML models on such hardware, which would be required if a model needs recalibration due to small fluctuations in the positioning of the recording electrodes. However, retraining the model is more challenging in this context as it requires more computational resources than using a pre-trained for inference, i.e., classifying brain activity. Petschenig et al. (2022) addressed this challenge by benchmarking algorithms for classifying whisker deflection amplitudes based on MEA recordings from the barrel cortex of an anesthetized rat. Among the benchmarked methods, the Liquid State Machine (LSM) algorithm turned out to be highly accurate, and this is particularly relevant since it implements a Spiking Neural Network (SNN) developed explicitly for neuromorphic hardware. Hardware of this type are small, brain-inspired computing architectures designed for processing brain signals in a highly efficient manner, representing an appropriate option for chronically implanted prostheses (Buccelli et al., 2019; Werner et al., 2016).

The barrel cortex has, therefore, served as an ideal experimental model for investigating which computational approaches are appropriate for a neural prosthesis. The above-summarized developments in the classification of the whisker stimulus intensity based on stimulus-evoked activity from the barrel cortex are highly promising for the field of neuroprosthetics, as they demonstrate the ability to implement BMIs or BMBIs in low-powered hardware and their capability to decode information from neural activity. However, these results are based on recordings under anesthesia, which is known to affect the pattern of neural activity (Steriade et al., 1993). Also, even in the awake state, cortical responses are known to differ depending on whether an animal is actively engaged in a sensory processing task or merely exposed to sensory stimulation without the need to react to it (Otazu et al., 2009; Carcea et al., 2017; De Franceschi and Barkat, 2021). Thus, it remains unclear if ML classifiers also perform well on data recorded from awake and behaving animals. Such data is expected to contain both a higher level of and stronger variability in spontaneous activity compared to the anesthetized state, and therefore, it could be more challenging for the algorithms to detect or classify stimulus-evoked activity. Moreover, whether such models can be transferred from one individual to another is unknown. Such generalizable models would allow for training models on larger datasets composed of recordings from more than one individual, making the models more robust.

In this study, we benchmark lightweight ML algorithms on the task of whisker stimulus detection based on an electrophysiological dataset obtained from the barrel cortex of four mice while they performed a behavioral task. Using recordings from several sessions with different mice allowed us to clearly separate data from one individual for model training and then use this data for model evaluation, providing a clear picture of how these models generalize across individuals.

Relevant to the application of such models, we benchmark their runtime and investigate the impact of including data from multiple cortical layers. For the Liquid State Machine, the fastest and one of the best-performing algorithms, we analyzed how detection accuracy changes for detecting stimuli with different intensities and shorter MEA recordings.

## Results

### Dataset

**Figure 1:**
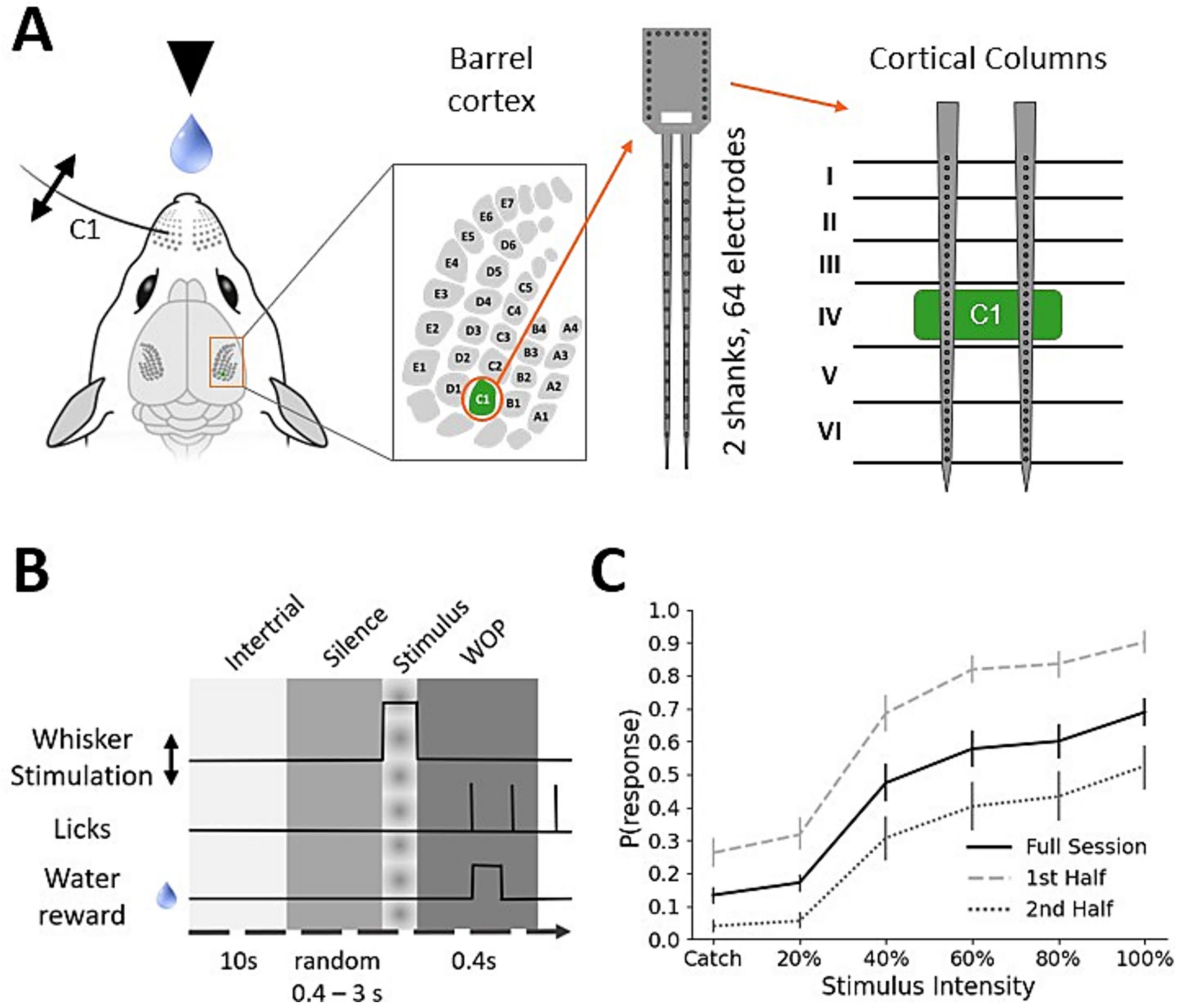
Experimental setup and psychometric curves. **(A)** Head-fixed mouse within reach of a water spout. Multi-electrode-array recordings are obtained from the cortical column associated with the stimulated whisker C1. A silicon probe with 64 electrodes, 32 on each of two shanks, was used to measure activity in all cortical layers, as shown in the sketch on the right-hand side panel, with barrels indicated in L4. For histological image examples, we refer to our previous experiments, in which shanks were only inserted after their location had been marked (Yeganeh et al., 2022). **(B)** Overview of the behavioral task. Mice were conditioned to respond to whisker stimulation by licking at the water spout. Responding to stimulus trials (but not catch trials) within 500 ms was rewarded with a drop of water. We refer to the time window from −400 ms to 0, relative to stimulus onset, as the pre-stimulus, or spontaneous, activity. The peri-stimulus or evoked activity is represented by the time window 0 to +100 ms as the stimulus is a sinusoidal whisker vibration of 100 ms duration. A hit trial, a stimulus trial with a successful mouse response, is defined as a lick within the window of opportunity (WOP) from +100 to +500 ms. Trials with licks between 0 and +100 ms were excluded as licking activity caused strong artifacts in the LFP. **(C)** Psychometric curve averaged across 19 sessions from 4 mice. Only trials that remain after all filtering criteria are applied are considered (see Methods). Whisker stimulus intensity ranged from 0% (no stimulus) over 20, 40, 60, and 80–100% intensity (percentage of maximum deflection amplitude). Trials without stimulus (0% intensity) are called catch trials and were included to estimate the response rates achieved by random licks. Licks within the WOP in catch trials are called false alarms. Error bars show the standard error of the mean (SEM) computed over sessions. Subpanels in A were modified from (Yamashita et al., 2018).

The dataset was obtained from the barrel cortex of four head-fixed water-restricted mice while performing a go/no-go whisker stimulus detection task. Mice were trained to respond to whisker stimulation by licking at a water spout (Figure 1A). During experimental sessions, a silicon probe with two shanks, 32 electrodes each, was used to obtain electrophysiological recordings from cortical layers (L) 2/3, 4, 5, and 6. Based on a current-source-density (CSD) analysis, we identified different cortical layers and assigned all electrodes to their corresponding layer, selecting the electrode that was most central to derive a layer-specific signal from the MEA recordings (see Methods). Using the signals from different layers allowed us to investigate which layer provides the most important information for stimulus detection and whether integrating the signals from all layers leads to more accurate detection.

In total, 19 experimental sessions were performed with 300 trials each. Each trial of the behavioral task consisted of different epochs (Figure 1B). Before whisker stimulation, the mice had to refrain from licking for at least 400 ms (silence period). Since stimuli were only presented when animals refrained from licking for at least 0.4–3 s (varying randomly from trial to trial), we were able to use the 400 ms before stimulus onset, called *pre-stimulus*, to characterize spontaneous activity in the barrel cortex. The stimulus itself consisted of a single-whisker 60-Hz sinusoidal vibration of 100 ms duration, delivered in the rostrocaudal direction to the C1 whisker. We used this *peri-stimulus* time window to analyze stimulus-evoked activity. If the mouse responded within 500 ms after stimulus onset, it was rewarded with a water droplet. Each recording session included 50 catch-trials without stimulation (0% intensity to assess spontaneous licking) and stimulus trials with different intensities ranging from 20 to 100% of maximum amplitude (corresponding to amplitudes between 93 and 463 μm and angular velocities ranging from 324 to 1,600 °/s at 2 mm from the whisker base, respectively; see Vandevelde et al., 2023, for more details). Stimuli in this range have been found to induce weak to moderately strong activity in barrel cortex and therefore span the range of barely to easily detectable stimuli (Yang et al., 2016; Vandevelde et al., 2023). Each stimulus was presented 50 times, and the stimulus sequence was randomized. The use of different intensities allowed us to investigate the response rate of mice during the behavioral experiment, as well as investigate the shape and strength of the evoked response in the barrel cortex. Using a randomized sequence was important to prevent mice from learning any patterns in whisker stimulation, which could potentially bias the response. The response rate increased monotonically with increasing stimulus intensity (Figure 1C), which is expected considering similar experiments (Stüttgen et al., 2006; Stüttgen and Schwarz, 2008; Stüttgen and Schwarz, 2010).

For more details about the behavioral experiment, we refer to our previous publication (Vandevelde et al., 2023).

### Setup of machine learning classifier benchmark

As we integrated data from different recording sessions, we could not use the spiking activity as each session provides a different set of single- and multi-units, which precludes the assembly of a common feature vector to describe trials across sessions, a prerequisite for the ML algorithms. Besides this, it has been demonstrated on a single-session dataset that (i) LFP features are as informative as features derived from multi-unit activity and (ii) it is not necessary to derive more sophisticated features from the LFP, such as the response peak amplitude or response onset latency, because using the raw signal provides sufficient information for the ML methods used (Petschenig et al., 2022). In general, the LFP recording is more stable in comparison to action potentials, and therefore, it is recommended to use the LFP in the context of neuroprosthetics (Andersen et al., 2004; Markowitz et al., 2011). Following these previous studies, we use the raw LFP traces at 1,000 Hz resolution, low-pass filtered at 150 Hz but without further feature engineering, referring to them as *RAW* features, hereafter. Additionally, we investigate *FFT* features, consisting of the frequency components we obtain by applying the Fast Fourier Transform to the filtered signal (Brigham, 1988). Including the FFT features was inspired by Sederberg et al. (2019), who demonstrated that field potentials in the barrel cortex can be informative with respect to how whisker stimuli are perceived in the barrel cortex of awake mice.

We chose to use the LFP for two purposes: first, stimulus detection (SD), i.e., to decode whether a stimulus was presented to the animal, and second, response prediction (RP), i.e., to predict whether the animal was responding to the whisker stimulus. To that end, we benchmarked six ML classification algorithms, also called *classifiers*, known for good performance in various applications (see Table 1 for an overview of the selected classifiers). The Decision Tree (DT) achieves short runtimes due to its simplicity (Loh, 2011). Compared to a single decision tree, Random Forest (RF), which makes use of an ensemble of decision trees, and XGBoost (XGB), which uses stacked decision trees, are known to provide more accurate models while maintaining low runtime requirements (Breiman, 2001; Chen and Guestrin, 2016). Similar characteristics are associated with generalized linear models (GLM) that are, once trained, very fast during inference as they rely on a linear combination of weighted variables, so the logistic regression, a GLM with underlying logit function, has been included in the benchmark (Kleinbaum and Klein, 2010). Interestingly, comparable studies investigating brain activity classification did not explore algorithms developed for time series classification. We, therefore, included ROCKET (RCKT), a classification algorithm dedicated to time series input (Dempster et al., 2020; Pantiskas et al., 2022). As mentioned above, spiking neural networks are promising due to their ability to run efficiently on low-powered neuromorphic hardware, which motivated the inclusion of the Liquid State Machine (LSM). More complex artificial neural network architectures, such as long short-term memory (LSTM), have not been explored due to their high computational requirements and because they did not provide better models in comparable studies (Petschenig et al., 2022).

**Table 1:** Overview of ML classifiers used in the benchmark.

Algorithm	Runtime requirements	Developed for time series	Application examples related to neuroprosthetics
DT	Low		Srimaharaj and Chaisricharoen (2021) and Chikkudu and Annamalai (2024)
RF	High		Bhadra et al. (2025) and Giri et al. (2024)
XGB	Medium		Tiwari and Chaturvedi (2019) and Khanday et al. (2024)
RCKT	Medium	✓	Menon et al. (2023)
GLM	Low		Giri et al. (2024) and Chikkudu and Annamalai (2024)
LSM	Low		Petschenig et al. (2022) and Behrenbeck et al. (2019)

**Figure 2:**
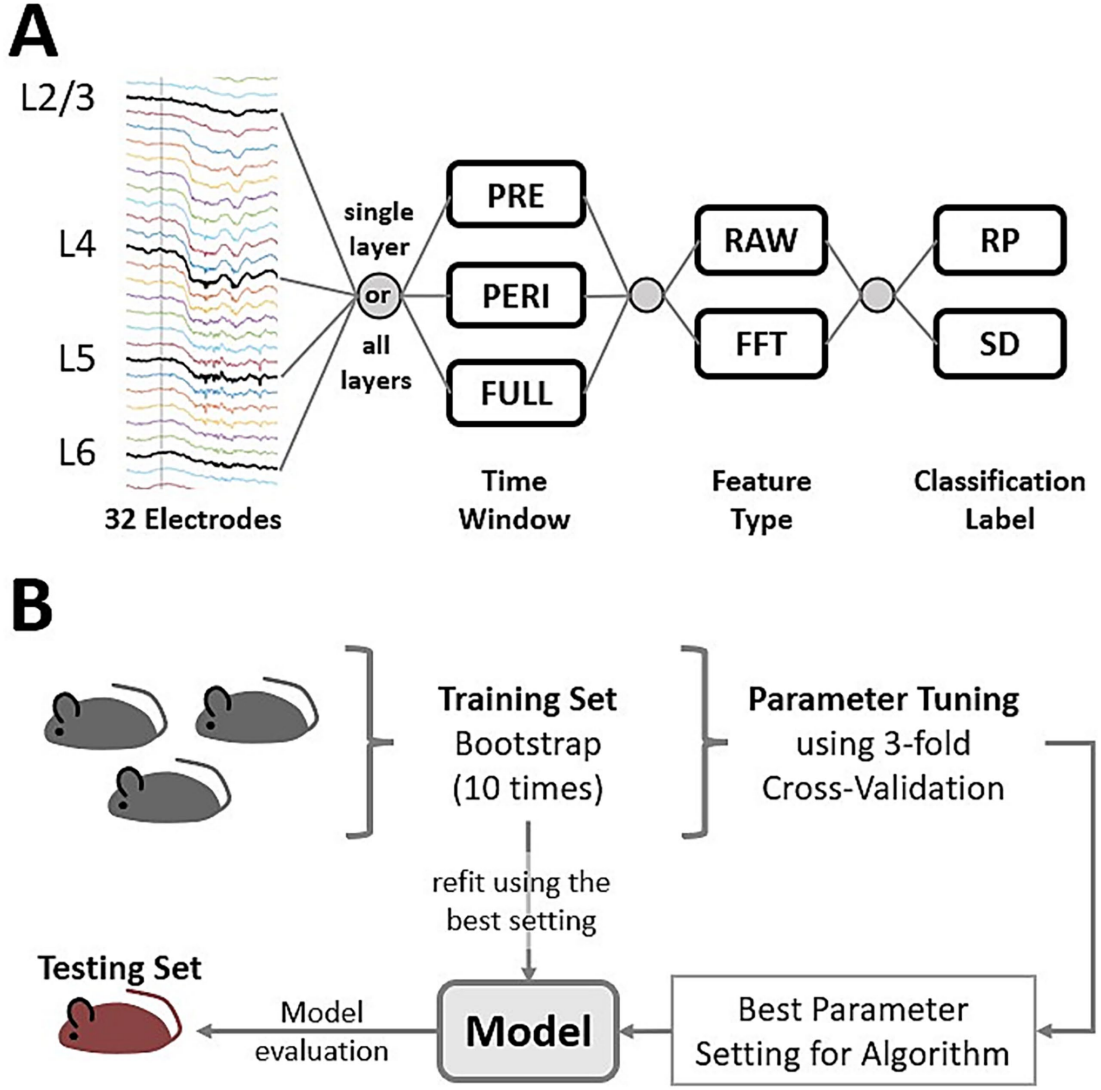
Machine learning features and setup of the benchmark. **(A)** Data flow for the machine learning classification scenarios. Four electrodes were selected to derive the LFP for different cortical layers. The time windows, PRE-stimulus, PERI-stimulus, or both (FULL), and the feature type specified the ML features. The classification label specifies the task of the algorithms, which was either response prediction (RP) or stimulus detection (SD), the latter being the focus of this study. Using different combinations of layer, time window, feature type, and classification label results in 24 different classification scenarios. **(B)** Essential for the benchmark was splitting experimental trials into training and testing sets. The training set was used to explore the hyperparameter settings of the machine learning algorithms. Given one algorithm, multiple hyperparameter settings were evaluated by a grid search using a three-fold cross-validation. The setting providing the best results in the grid search was used to train a model based on the full training set and eventually validated using the testing set. The testing set was created using data from only one mouse to ensure that trials from the same individual or experimental session were not included in both training and testing sets. This was repeated for four mice. Given one training–testing split, 10 bootstrap samples were drawn from the training set to train and evaluate multiple models. Given one classification scenario and one algorithm, this results in 40 model evaluations, increasing the statistical power of aggregated measurements for model accuracy. Feature values for RAW and FFT were linearly scaled using Min-Max Scaling (scaling values to the range [0, 1]). The minimum and maximum were computed from the training set only, then used to scale feature values in the training and testing sets.

All algorithms were compared based on different classification scenarios. The term *classification scenario* is here used to describe a specific combination of which classification task (SD or RP) is considered and which features are used, further specifying the scenario by type (RAW, FFT), cortical layer, and time window from which the recording was derived (Figure 2A). For each algorithm, hyperparameter tuning was applied by evaluating a grid of parameter settings, also called grid search, using 3-fold cross-validation on the training data (see Supplementary Table T1 for details). Furthermore, the model evaluation was carefully done, splitting up experimental trials based on individual mice to ensure cross-individual validation in a way that trials from one individual are separated by the training set, used for hyperparameter tuning and training the classification model and testing set, used to evaluate the model performance (Figure 2B). Training–testing splits have been repeated four times, using four individuals and for each split, 10 bootstraps were drawn to increase the reliability of the aggregated accuracy measures. For each bootstrapping, the size of the training and testing set differs because the number of experimental sessions available differs between mice (see Supplementary Table T2 for details about the set sizes).

In all classification scenarios, we used perfectly balanced datasets, meaning each subset contained the same number of positive trials (stimulus trial or response trial) and negative trials (catch trial or no-response). This was achieved by down-sampling trials from the overrepresented, and due to the expected 50% positives in the data, we can assume that the baseline accuracy achieved by random predictions converges to 50%. Note that including some scenarios was mainly motivated by creating a baseline scenario in which a low accuracy (close to 50%) is expected. For instance, we would not expect pre-stimulus LFPs to encode information useful for stimulus detection. Hence, an accuracy of 50% would be expected for this scenario from any classifier. For response prediction, the expectation can be slightly different. Even though the barrel cortex is not involved in decision-making, its spontaneous activity might encode different states related to attention and excitement, which is relevant to the behavioral experiment (Cowley et al., 2020). Hence, it might be possible for classifiers to leverage weak patterns predictive for the response, even from pre-stimulus (spontaneous) activity.

### Machine learning classifier benchmark

The first analysis addresses the question of the extent to which ML classification algorithms, also called *classifiers*, can leverage information from LFP features to train accurate classification models for response prediction (RP) or stimulus detection (SD) and which classifier trains the most accurate models.

**Figure 3:**
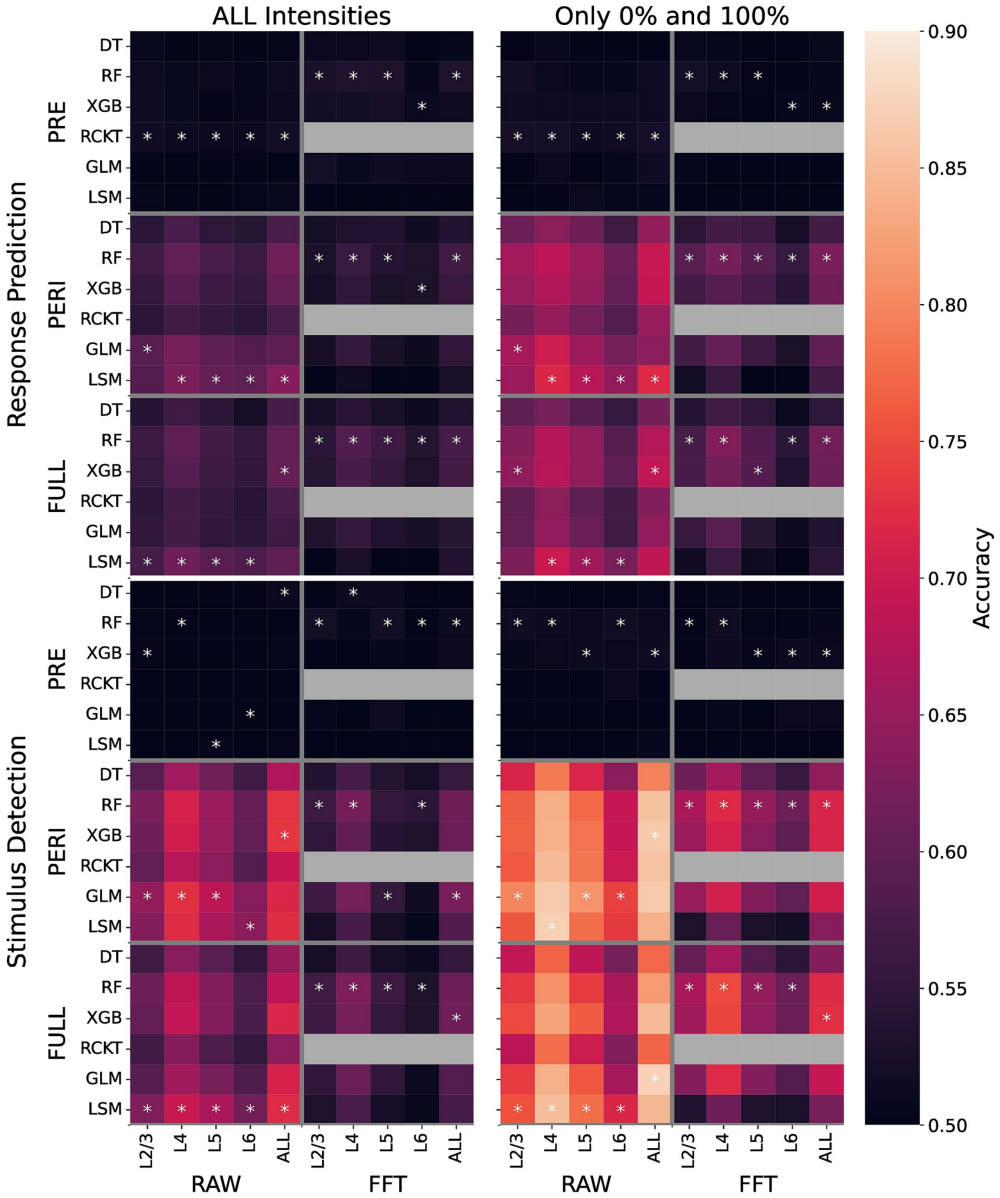
Machine learning benchmark. Accuracy represents the classification performance of six different algorithms in several supervised classification scenarios, mainly defined by the task of stimulus detection (SD) and response prediction (RP). These are specified by the time window before (PRE) or after (PERI) stimulus onset or the complete time window (FULL), concatenating the PRE- and PERI-stimulus LFP signal. Furthermore, there are different feature types, RAW and FFT, which were derived from the LFP measured in different cortical layers (L2/3, L4, L5, L6) or using a concatenation of feature vectors from all layers (ALL). The underlying datasets contain either trials from all intensities or only catch trials (0%) and full intensity (100%) trials. The asterisks indicate which classifier achieved the best accuracy.

Figure 3 provides an overview of the accuracy obtained for all classifiers (innermost *y*-axis) applied to the classification scenarios, specified by the two classification tasks (SD, RP), the time window for the recording (PRE, PREI, FULL), feature type (RAW, FFT), cortical layers (innermost *x*-axis), and the stimulus intensities used for trial selection (see subtitles). Brighter colors represent higher accuracy observed for SD in general. Using the PERI and FULL time window results in higher accuracy for both tasks, SD and PR. Regarding the feature types, the RAW features are more informative than the FFT, shown by higher accuracy for both SD and RP. The lower half of the heatmap shows accuracy measures obtained for SD, for which a higher level of accuracy can be observed in general. The accuracy for SD reached up to 90% when trials with 0% (catch-trials) and maximum stimulus intensity of 100% were contrasted.

Features derived from L4 are more informative than those derived from other layers. Considering a feature vector combining all layers (ALL on the *x*-axis) slightly improves the accuracy over the performance of L4 alone. As expected, the peri-stimulus activity (PERI), describing the stimulus-evoked response in the LFP, is more informative for both classification tasks in comparison to the pre-stimulus activity. Classification models trained on pre-stimulus activity features are unable to perform accurately for SD, as expected. For RP, however, the accuracy slightly increased over the 50% baseline when RF is used on pre-stimulus FFT features (Figure 3).

Regarding the classifiers, RF performs well in scenarios exploring FFT features. For the RAW features that are more informative for the SD task, we observed that the GLM and LSM achieved the highest performance, with RCKT slightly lower. We do not see an improvement in model performance for SD when integrating the FULL time window over using peri-stimulus alone (Figure 3).

However, from the median accuracies discussed in this section, it is not clear if it is worth integrating all layers, expected to increase model training time, over using solely L4. Moreover, a runtime analysis is required for the best algorithms, considering the practical application of these models. Therefore, a more detailed comparison of the most accurate algorithms follows, focusing on SD based on the peri-stimulus RAW features.

### Detailed comparison of the most accurate algorithms

**Figure 4:**
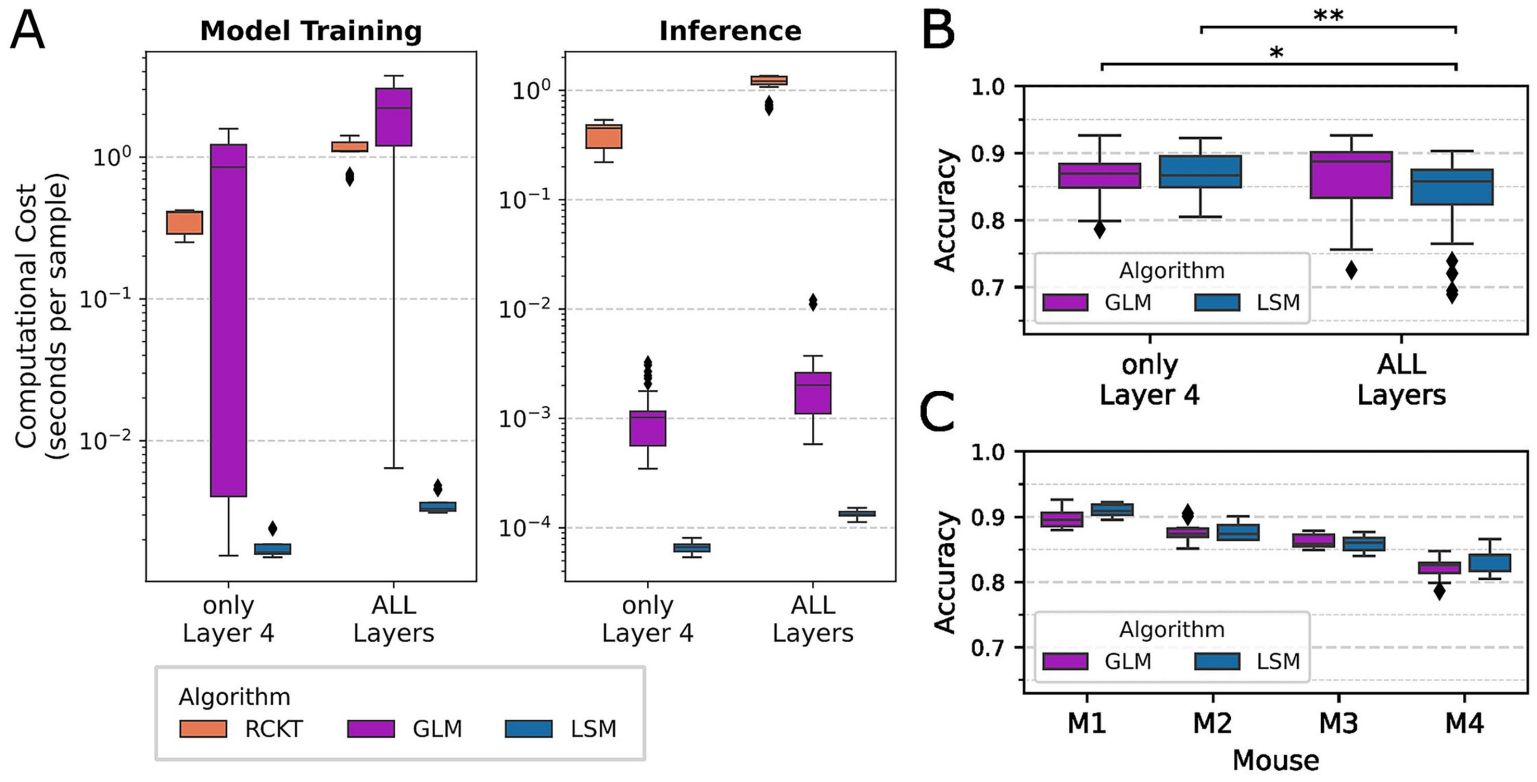
More detailed comparison of the most accurate and fastest algorithms. The scenario selected for these detailed visualizations is the SD using RAW features based on 0 and 100% intensity trials only. **(A)** The computational time was measured during model training and when a trained model was applied to samples from the testing set to classify those (inference). As training and testing sets differ in size depending on which mouse was used to produce the split, the runtime, measured in seconds, was normalized using the size of the training and testing sets, respectively. Note that the range of the *y*-axis differs between the subplots and that the *y*-axis is on a logarithmic scale. **(B)** Comparison of the accuracy distribution achieved by GLM and LSM using the raw LFP (see RAW in Figure 3) derived from layer L4 or concatenating all layers. The asterisks indicate significant differences with * →*p* value < 0.05 and ** →*p* value < 0.01 using the Wilcoxon signed-rank test. Distributions are not significantly different if nothing is indicated. **(C)** Classification performance of GLM and LSM for the different training, testing splits based on four mice. Boxplots describe the distribution of accuracies measured on 10 bootstrap samples. Diamonds show the outliers of the boxplot.

For this comparison of the best-performing classifiers, the SD classification task has been selected with 0 and 100% intensity trials only, because the highest classification accuracy was achieved in this scenario. Considering GLM, RCKT, and LSM as the top-performing classifiers in terms of accuracy, the LSM is the strongest competitor based on the runtime comparison (Figure 4A). RCKT was faster than the GLM for model training but much slower during inference. In general, the runtime for model training and inference was higher when all layers were used, which is expected as algorithms are processing four times more ML features than using only a single layer. Focusing on LSM and GLM as the fastest algorithms, we investigated the difference in accuracy for the SD task using all layers and only L4 (Figure 4B). In this comparison, the LSM achieved the lowest performance when all layers were integrated, which suggests that the GLM is more efficient in dealing with high-dimensional data as it achieved the highest median accuracy overall when it integrated all layers. However, as the accuracy achieved by the GLM with all layers is not significantly higher than that of the LSM with only L4, we conclude that the LSM is the best choice for stimulus detection, especially due to its superior performance in the runtime comparison with the lowest model training and inference runtimes using only L4. Furthermore, it achieved slightly more robust accuracy distributions across all individuals than the GLM, for which more outliers and lower median accuracy measures were observed overall (Figure 4C). To assess whether the accuracy scores of the GLM and LSM (Figure 4B) differed significantly, the Wilcoxon signed-rank test was applied on 40 pairs (four mice times ten bootstraps). A pair of values consists of the accuracy values from the two classifiers, obtained on the same mouse-specific testing set and the same bootstrap sample, using random seeds to ensure bootstrap samples are equal.

### Stimulus detection for different intensities using shorter peri-stimulus LFP signals

For practical applications, it is important to know how accurate the detection is for stimuli of different intensities (Petschenig et al., 2022; Wang et al., 2018b). Furthermore, it is crucial to investigate how long the LFP recordings need to be to maintain accuracy at the highest level possible. The shorter this recording time is, the earlier the model can be applied after stimulus onset. To address these two aspects, an additional SD experiment was performed using subsets of the data with catch-trials and stimulus trials of intensities from 20 to 100%. Note that this includes the results from the benchmark obtained for 100% stimulus intensity and the complete 100 ms of peri-stimulus activity. These SD experiments were further specified by different lengths of the RAW LFP signal as input for the classification model, using L4 as the most informative layer. All additional classification experiments were done for the LSM using the validation strategy applied during the initial ML benchmark analysis (Figure 2B).

**Figure 5:**
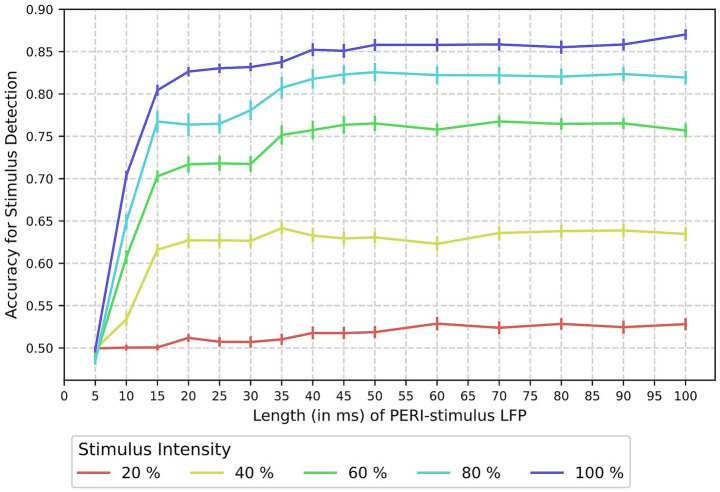
Accuracy of stimulus detection for different intensities using shorter peri-stimulus LFP signals. Accuracy for stimulus detection when the LSM is trained and evaluated to differentiate catch trials (0% intensity) from stimulus trials of different intensities (20–100%). Each classification experiment was repeated using shorter LFP recordings derived from the peri-stimulus signal, as shown on the *x*-axis. The lines describe the mean accuracy based on 40 training–testing splits, and the error bars describe the standard error of the mean.

We observed that LSM’s detection accuracy is lower for stimuli of low intensities (Figure 5). In comparison to the accuracy achieved on the complete (100 ms) peri-stimulus LFP, approximately 35–40 ms is required to achieve a high level of accuracy for the higher intensities greater than or equal to 60%. For 40% intensity, even though the overall accuracy was much lower than for higher intensities, the LFP can be as short as 15 ms to achieve high accuracy for this case. Note that the RAW features were used for these tests, representing the LFP after low-pass and band-stop filtering, which further delays this process by a total of 0.2 ms (measured on a common CPU, expected to be slightly higher on neuromorphic hardware).

### Imbalanced data and model calibration

To mitigate potential impacts of class imbalance (over- or underrepresentation of the positive class), datasets have been downsampled to train models on data with 50% positive (stimulus present) and 50% negative (stimulus absent) samples. For deployment, however, it is important to investigate how algorithms perform on imbalanced data. Using L4 as the most informative layer and RAW as the most informative feature type for stimulus detection, we benchmarked the GLM and LSM again and added comparisons on data without downsampling. We used the dataset with all stimulus intensities, resulting in 83% stimulus trials, and the dataset containing only 0% stimulus intensity (catch) trials and 100% stimulus intensity trials, for which downsampling had almost no impact, as the fraction of positives changed from 50 to 49.3%. Due to the class imbalance in the data, we added ROC and Precision-Recall curves to evaluate model performance (Saito and Rehmsmeier, 2015). Additionally, we used the Brier loss score to evaluate model calibration and studied the impact of Platt Calibration on the model probabilities from GLM and LSM (Platt, 1999; Niculescu-Mizil and Caruana, 2005).

The results from this analysis (see Supplementary Figure S1) show that (i) the GLM is well-calibrated and does not benefit from Platt Calibration, (ii) the LSM needs Platt Calibration to improve its calibration performance, especially for imbalanced data, and (iii) the overall performance of GLM and LSM is very similar, both achieving area under ROC and Precision-Recall curve clearly better than a random guess or naïve model.

Note that testing data is not used for Platt Calibration. A default Logistic Regression (scikit-learn) is trained as a calibration model on the classification probabilities and class labels of a 33% hold-out dataset from the training set. Consequently, the model evaluated on the mouse-specific testing set has been fitted on only 67% of the training set when using Platt Calibration. The fitted calibration model is applied to transform the classification probabilities from the testing set before computing the evaluation metrics.

## Discussion

Three ML classification algorithms were identified as the best-performing ones, achieving the highest accuracy for stimulus detection. In a subsequential runtime analysis, the LSM has been superior in achieving the lowest runtimes for model training and inference. Hence, the LSM has not only been faster within the runtime analysis performed on an ordinary CPU but it is also expected to be more efficient when deployed on neuromorphic hardware as it, in contrast to other machine learning algorithms, mirrors the physical architecture of low-powered chips used in such hardware. Accordingly, the LSM could even be used to train a model on the prosthesis, which is essential for recalibrating a model by retraining after it has been implanted (Petschenig et al., 2022). Recalibration of a model can be necessary if the electrodes measuring the input signal are slightly moved and small shifts in the signal are expected.

Furthermore, the results of this study show that the classification models are robust across four mice from which the data was derived, with the LSM being slightly more robust than the GLM. This is promising as models could potentially be trained on data collected from a large cohort of patients and then deployed in new patients. When the model is used for classification on low-powered hardware, the low inference times are paramount. While the LSM and GLM were much faster when applying the model (inference) than training it, the ROCKET classifier showed similar runtime requirements during training and inference. This is explained by ROCKET’s algorithmic strategy of applying convolutional kernels to the time series signal before using a sample as input. This signal preprocessing is required during training and when a trained model is used. Hence, this requires additional computational resources during inference.

The runtime analysis also showed that all algorithms require less computational resources when only one layer is used. Even though the GLM achieved the highest stimulus detection accuracy when the signal of all layers was integrated, its accuracy was not significantly higher than that of the LSM when applied with only L4. Observing L4 as the most informative layer within the barrel cortex is not unexpected, as it is the main recipient of the thalamic input about whisker deflection, and multi-unit activity in this layer features higher neurometric detection sensitivity than in other layers (Vandevelde et al., 2023; Reyes-Puerta et al., 2015; Reyes-Puerta et al., 2015). The signal is expected to become noisier as it is forwarded to other layers of barrel cortex, which also receive information from other cortical areas, and may integrate this information with texture information provided by consecutive whisker-object contacts for routing to action-related cortical areas (Zuo and Diamond, 2019; Gămănuţ et al., 2018). The redundancy of whisker information represented in different cortical layers, along with (apparent) noise added from other brain areas, could explain why the LFP in layers other than L4 does not strongly enhance stimulus detection (Arabzadeh et al., 2006). Additionally, using a concatenation of feature vectors from all layers results in high-dimensional data, characterized by a high number of features but a low sample size, which can lead to less accurate stimulus detection models, as ML algorithms are less effective in capturing meaningful patterns in the large number of features (Horenko, 2020; Vecchi et al., 2022). We conclude that layers other than L4 can provide additional information to slightly improve the SD accuracy, as shown by the GLM, but that these improvements are too small to justify choosing the GLM over the LSM, being much faster and only slightly less accurate, when using solely L4.

Besides the runtime of the machine learning model itself, it is also important to know how long the LFP signal needs to be for accurate stimulus detection. We, therefore, analyzed the classification performance for stimulus detection using shorter RAW feature vectors and found that high accuracy, as observed for 100 ms of the peri-stimulus signal, can be maintained using only 35–40 ms of the signal. For practical applications, this means that a delay by this time frame should be expected before a stimulus can be detected and processed in a BMI or BMBI device. This analysis has also been done for different intensities and reveals that accuracy decreases with decreasing intensity. This is a critical observation, indicating that only strong stimuli are detected reliably. At the same time, it reflects the perception of mice as they also showed a lower response rate for weaker stimuli during the behavioral experiment (Figure 1C), and because generally, whisker stimulus intensities were relatively low compared to the full range of possible contacts during unrestrained locomotion (Ritt et al., 2008).

Generally, an LFP signal describing the evoked response of a whisker stimulus in the barrel cortex is characterized by a strong drop in the current approximately 5–10 ms post-stimulus onset (see example sessions in Supplementary Figure S2). This change in the signal seems to be strong and consistent enough that a classifier does not need to leverage complex, non-linear patterns, which is implied by the strong performance of the linear model (GLM) used in the benchmark. This might also explain why the RAW LFP signal is more informative than oscillatory patterns encoded by the FFT features. Comparing the LFP and FFT features of two sessions from our dataset revealed an increased amplitude for the frequency components describing 60 Hz, and its harmonics can exist (Supplementary Figure S2). However, these FFT patterns are inconsistent and only present if the electrode has been close to a cluster of velocity-sensitive neurons activated by the peak velocity for the whisker movement during stimulation. Remember that the vibrotactile stimulus was a cosine of 60 Hz, moving the whisker in the anterior–posterior direction. Importantly, in the primary afferent neurons of the trigeminal ganglion (the first station of the ascending whisker sensory pathway), activity in response to whisker deflection is strongly determined by stimulus velocity (Stüttgen et al., 2006). This is also true for whisker-responsive neurons in the thalamus (Temereanca and Simons, 2003) and barrel cortex (Pinto et al., 2000).

### Response prediction and the patterns in spontaneous activity

Besides investigating neural activity with respect to how whisker stimuli are processed in the barrel cortex, the dataset allowed us to search for patterns predictive of the behavioral response. This is a critical aspect as we record from a somatosensory brain region believed to be involved in the process of evaluating an incoming stimulus and provide the basis for sensory-driven decision-making (Staiger and Petersen, 2021). Considering the response prediction analysis as part of the ML benchmark, we observe a moderate accuracy for response prediction models trained on the RAW features from the peri-stimulus or full signal (Figure 3). However, an additional analysis based on the predictions of the corresponding models strongly suggests that the response prediction models actually perform stimulus detection, which results in quite accurate models due to the strong correlation between the presence and absence of stimulus and the occurrence of behavioral response (see Supplementary Figure S3).

Interestingly, our benchmark revealed that patterns exist in the harmonic oscillations derived from spontaneous (pre-stimulus) activity that are slightly predictive toward the response of mice (see RP with pre-stimulus FFT features, all intensities, Figure 3). For the FFT features derived from L2/3, L4 or L5 we observed accuracy slightly, but significantly better than the random baseline when all stimulus intensities are considered in the data (*p*-value from Fischer’s test < 1e^−20^ in all three cases). Even though this difference in accuracy is small, these results indicate that neuronal activity in the barrel cortex is also related to attention or anticipation of the expected whisker stimulus (Lee and Dan, 2012; Stüttgen and Schwarz, 2018). Such weak patterns in the spontaneous activity could reflect brain states in which the mouse is more attentive to perceive the stimulus, which impacts the response, especially when not only the full-intensity trials but also those with a small or intermediate intensity are analyzed. While these are indications relevant to the role of the barrel cortex in the behavioral response of mice, future work is required to further investigate the FFT features, which is out of scope for this study and might require further behavioral experiments.

### Practical application and limitations

Previous *in vivo* recordings in the rat barrel cortex have identified inhibitory interneurons in L4 as the units carrying the highest amount of sensory stimulus-related information (Reyes-Puerta et al., 2015; Reyes-Puerta et al., 2015). Thus, neuronal activity in L4 represents a good target to obtain information on the spatio-temporal properties of the sensory input. Measuring the activity from L4 is not only possible but also very reliable with invasive technologies as used in our experimental model. However, in a practical application, it will be more beneficial to use non-invasive technologies to avoid an intervention in the patient’s brain. Resolving activity in a specific portion of tissue without having direct access could be realized by laminar inference based on MEG recordings, although MEG is not applicable in neuroprosthetics (Bonaiuto et al., 2018).

Our study confirms that ML models can be highly accurate on whisker stimulus classification, even for LFP signals recorded from awake mice. However, the datasets used for such explorations, including the one we used, are based on precisely controlled single-whisker stimuli, certainly different from how whiskers bend and move when animals are actively palpating during unrestrained exploration (Petschenig et al., 2022; Wang et al., 2018a; Wang et al., 2018b). Therefore, for future work, it would be relevant to apply and test the suggested algorithms on recordings from microchips implanted in freely moving animals. The hardware required to do this could use wireless and battery-free devices (Martinez et al., 2018; Won et al., 2023). Recordings from such experiments might result in LFP traces that are less distinguishable, as we would expect that whisker deflections from freely moving animals are less distinct.

Additionally, our benchmark for imbalanced data covers only the case of overrepresentation of the positive class, which is the opposite of a real-life scenario in which stimulus events are less frequent compared to non-stimulus recordings. Our dataset provided well-defined trials for events with an induced stimulus, with five different intensities over catch trials without a stimulus. Thus, using all trials of the dataset without downsampling results in overrepresentation of the positive class. Nevertheless, this analysis demonstrates that both algorithms perform well on imbalanced data in general and reveals that additional calibration of classification probabilities is required when using the LSM, particularly on imbalanced data. Interestingly, on the imbalanced data, the GLM and LSM achieved accuracy values close to those expected from a naïve model that always returns the positive class (stimulus present). However, both algorithms yielded high area-under-the-curve scores when using the ROC and Precision-Recall curves as threshold-independent evaluation metrics. We therefore conclude that, in imbalanced deployment settings, both algorithms require decision threshold optimization, which is left for future work.

To realize the closed-loop system, the output of the ML classifier could be used to control an electrode located in another brain region to induce intracortical microstimulation that can convey artificial signals that may be as complex as the somatic perception of tactile properties from different objects (Jackson et al., 2006; O’Doherty et al., 2011). However, a complete experimental setup would be required to verify whether the whole process of reading in the MEA recording, applying the ML classifier, and creating a feedback signal is fast enough to restore brain functionality, establishing timely signal processing with other cortical areas.

### Model robustness and generalizability

Another important aspect is the robustness of the findings concluded from our ML-based analyses. The sample size is a key factor in statistical analyses, and machine learning models tend to be more meaningful and generalizable when trained on large datasets (Carlini and Wagner, 2017; Rice et al., 2020). Even though electrophysiological recordings are challenging from different perspectives, we could explore a comparatively large dataset using >3,400 trials for six stimulus intensities. The final subsets used for the ML-based analysis were restricted by a selection of trials for the different intensities, resulting in a sample size of at least 600 trials (see Supplementary Table T2), which is approximately three times higher than in comparable studies using only one recording session, e.g., (Petschenig et al., 2022; Wang et al., 2018a; Wang et al., 2018b).

In summary, based on a large electrophysiological dataset enabling a robust model evaluation, we demonstrated that SNNs are accurate and efficient in interpreting neural activity toward neuroprosthetics applications related to the barrel cortex. Using the LSM classifier, model training could be realized on a small microchip to be implemented on the prosthesis, which can be beneficial to retrain a model for adjusting to small drifts of the implanted electrode, for instance. While our tests were done on an infrastructure with common CPUs with the main perspective to compare the model training time between the different classifiers, it has been shown recently that LSMs can efficiently be implemented and executed on microchips, as demonstrated on a DYNAP-SE neuromorphic processor (Petschenig et al., 2022). Another opportunity is to train a model on an external machine, transferring the model and only applying it on the prosthesis (Wang et al., 2018b). If future technologies enabled a more stable implantation of the electrodes, retraining on the prosthesis might not be necessary. Our generalizability analysis confirms that using a pre-trained model is possible even if the underlying data was derived from an individual different from the patient who needs the prosthesis.

## Methods

### Electrophysiological dataset

The data investigated for this study were obtained from experiments with head-fixed mice performing a go/no-go whisker stimulus detection task (Vandevelde et al., 2023). Mice were water-restricted and learned that they were rewarded by a drop of water if licking within 500 ms after the onset of a 100-ms sinusoidal vibration of a single whisker. Before mice were trained to perform this task, they were habituated to the head-fixed setting, which was important to enable the recording of neural activity in the cortical column in the barrel cortex associated with the stimulated whisker. This was done with multi-electrode-array (MEA) silicon probes through all cortical layers using 2-shank-64-channel probes with a distance between the shanks of either 200 or 250 μm. (Neuro Nexus, Ann Arbor, United States, or Cambridge Neurotech, Cambridge, United Kingdom, respectively). Each shank has 32 electrodes, and the distance between electrodes is 25 μm, which enables the observation of neural activity over an extent of ~0.8 mm.

For more details about the mouse line, behavioral task training, habituation, surgery, MEA recordings, and the layer-electrode association using current source density (CSD) plots, we refer to Vandevelde et al. (2023). After inspecting the CSD plots, we used one electrode per layer to derive layer-specific LFPs from the MEA recordings. The location and extent of layer 4 could be easily identified by a prominent short-latency current sink in the CSD, computed from the average LFP response to repeated high-intensity C1 whisker deflections, and therefore could be separated from layers 2/3 above and layer 5 below. The border between layers 5 and 6 was estimated based on recording depth (for more details, see Vandevelde et al., 2023, as well as Reyes-Puerta et al., 2015; Yang et al., 2017, 2018). Psychometric curves (Figure 1C) differ slightly from those shown in our previous study (Vandevelde et al., 2023) because we excluded trials with a lick response within the first 100 ms after stimulus onset as the muscle movement related to licking caused artifacts in the recordings.

### LFP preprocessing and LFP features

To exclude LFP traces contaminated by artifacts, we applied trial filters for the full range from −420 ms to +120 ms according to the following criteria. Trials in which animals were presumably moving and/or licking randomly had large voltage fluctuations and sometimes burst-like oscillations in activity. Trials in which the signal reached saturation were identified by applying a cutoff of ±2 mV at any time point within the time range specified above. Bursts were identified by moving time windows of 20 ms in which all electrodes strongly correlated, computed by Pearson’s correlation (Schober et al., 2018). Electric shorts were partly captured by the MEA electrodes, resulting in a continuous 50 Hz sinusoidal signal, and trials with a strong 50 Hz frequency power were removed. Trials for which a lick was detected within the above-mentioned time window were also excluded.

The RAW features describe the LFP trace at 1,000 Hz resolution, resulting in 400 features describing, for instance, the pre-stimulus activity of 400 ms from −400 ms to 0 ms (stimulus onset). Hence, the peri-stimulus activity from 0 to +100 ms is described by a feature vector of length 100, while 500 features represent the full time window from −400 ms to +100 ms. Using causal Butterworth filtering, the LFP traces were low-pass filtered at 150 Hz, and the 47–53 Hz band was removed, using a first-order low-pass and a sixth-order band-stop filter (Selesnick and Burrus, 2002). Using the same time-windows, the Fast-Fourier-Transform (FFT) was applied to derive the strength of the frequency components (Brigham, 1988). According to the length of the signal derived from the investigated time windows, the resolution is 2.5 Hz and 10 Hz going up to 147.5 Hz and 140 Hz for the pre- and peri-stimulus activity, respectively. The first FFT feature (0 Hz) represents the constant, which is simply the average over the signal. Eventually, 60 pre-stimulus and 15 peri-stimulus features describe the FFT feature vectors, resulting in 75 features for the full range. Due to the strong differences between ongoing and evoked activity, the FFT was applied to those time windows separately, and the resulting FFT features were merged afterward in order to create the full (pre- and peri-stimulus) FFT feature vector.

### Soft- and hardware specifications

We used MOGON II, the high-performance computing system from the University of Mainz. The whole analysis was implemented in Python. We ran DT, RF, and GLM from the *scikit-learn* package (Pedregosa et al., 2011) and XGB using a separate library called *xgboost* (Chen and Guestrin, 2016). The algorithm ROCKET (RCKT) was integrated via the *sktime* package (Löning et al., 2019), implementing its univariate and multivariate variants (Dempster et al., 2020; Pantiskas et al., 2022). The LSM algorithm was used from the GitHub repository https://github.com/IGITUGraz/LSM, published by Maass et al. (2002). The CPU specifications on the SMP compute nodes on MOGON are Intel® Xeon® E5 v4, 2.2GHz.

## Data Availability

The datasets used, and the Python code performing the ML benchmark are publicly available in a gitlab repository: https://gitlab.rlp.net/salbrec/sdrpml.git.
