# Supplementary material for: Spiking neural networks provide accurate and time-efficient models for whisker stimulus classification of the awake mouse
Source: Front Neurosci. 2026 Apr 1;20:1605209. doi: 10.3389/fnins.2026.1605209 (PMC13079647; doi:10.3389/fnins.2026.1605209)
Supplement: Supplementary file 1 [file Supplementary_file_1.pdf]

Supplementary Material

Spiking neural networks provide accurate and time-efficient models for whisker stimulus classification of the awake mouse

Steffen Albrecht, Jens R. Vandevelde, Edoardo Vecchi, Gabriele Berra, Davide Bassetti, Maik C. Stüttgen, Heiko J. Luhmann, Illia Horenko

**Algorithm**	**Parameter Grid**
Decision Tree (DT)	*max_depth*: 3,4,5,6<br>*min_samples_split*: 2, 5, 10<br>*min_samples_leaf*: 1, 2, 5, 10<br>*criterion*: gini, entropy
Random Forest (RF)	*max_samples*: 0.5, 0.75<br>*min_samples_split*: 2, 5, 10<br>*min_samples_leaf*: 1, 2, 5, 10<br>*criterion*: gini, entropy
XGBoost (XGB)	*num_boost_round:* 100, 250<br>*eta*: 0.1, 0.3<br>*max_depth*: 6, 9, 12<br>*subsample*: 0.9, 1.0<br>*colsample_bytree*: 0.9, 1.0
Rocket (RCKT)	*num_kernels:* 500, 1000<br>*max_dilations_per_kernel:* 2,8,16,32,64<br>*n_features_per_kernel:* 2,4,6,8
Generalized Linear Model (GLM) – Logistic Regression	**General parameters**:<br>*C*: $10e^{x}$ with $x \in \mathbb{N}$ ranging from -5 to 5 in steps of size 1<br>**Solver used depending on penalty:**<br>*no penalty: lbfgs*<br>*l1 penalty: liblinear*<br>*l2 penalty: lbfgs*<br>*elasticnet: saga*
Liquid State Machine (LSM)	*excitatory neurons*: 500, 1000, 1500<br>*inhibitory neurons*: 100, 250, 400<br>*recurrent neurons*: 250, 500, 750<br>*regularization*: 1, 2, 10

Table T1 – Parameters for Hyperparameter Tuning

Size of Training Sets

	All Intensities		Only 0% and 100%	
	**SD**	**RP**	**SD**	**RP**
Mouse M1	976	2530	934	798
Mouse M2	1002	2490	970	788
Mouse M3	662	1782	648	552
Mouse M4	1074	2414	1024	772

Size of Testing Sets

	All Intensities		Only 0% and 100%	
	**SD**	**RP**	**SD**	**RP**
Mouse M1	262	545	258	172
Mouse M2	236	582	222	182
Mouse M3	576	1290	544	418
Mouse M4	164	330	164	134

Table T2 – Sample sizes for training/testing sets in different scenarios

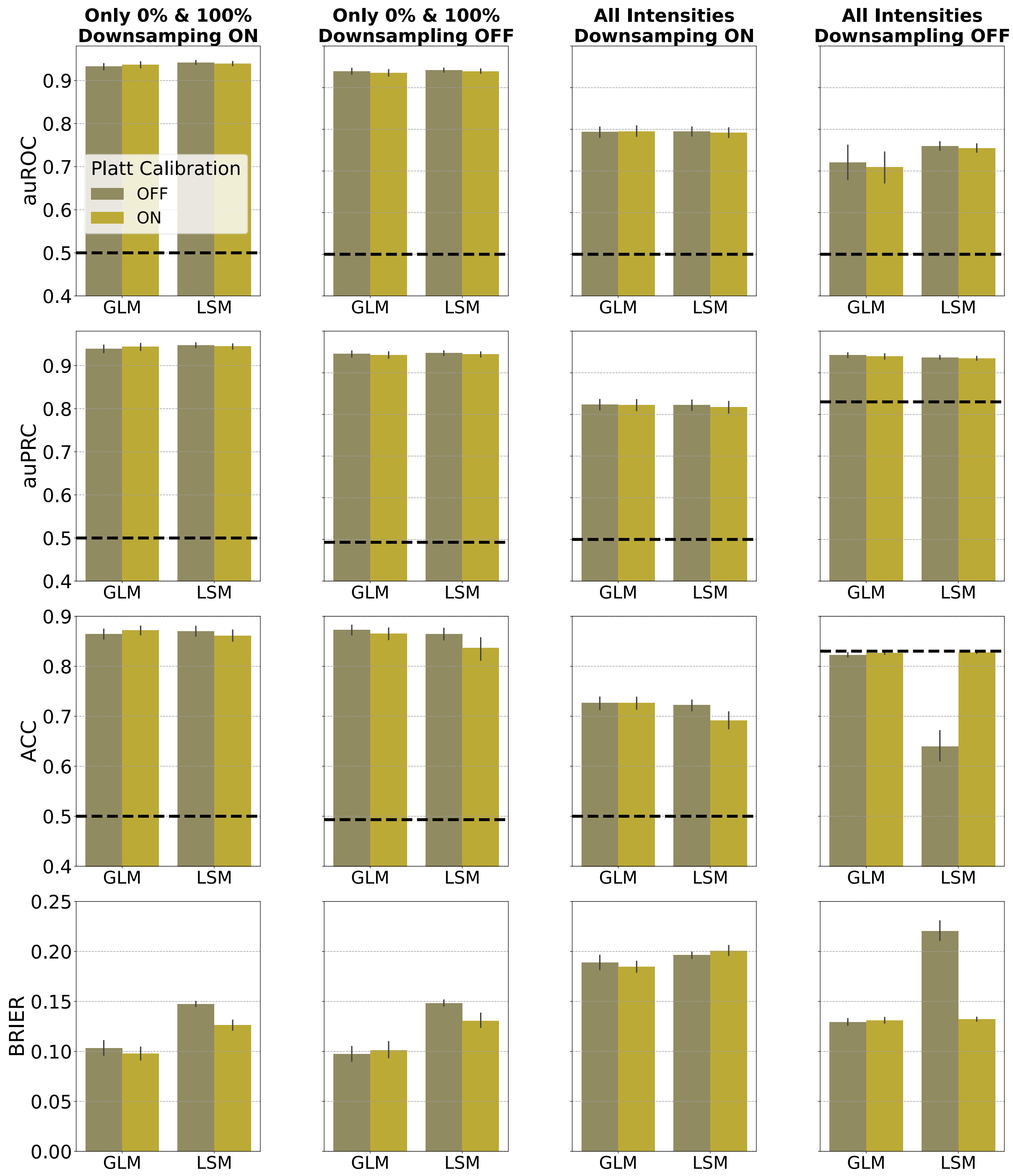

Figure S1 – Imbalanced Data and Model Calibration

Barcharts showing the classification performance (auROC for area under ROC curve, auPRC for area under Precision-Recall curve, ACC for accuracy) and calibration performance (BRIER Loss Score) of the GLM and LSM in two different scenarios with and without downsampling. Additional tests have been applied to investigate the impact of Platt Calibration; see the colors of the bars. The black dashed lines represent the performance of a random-guess model or naïve model that always returns the positive class label: 0.5 for the auROC and the fraction of positives for the ACC and auPRC. Error bars show the 95% confidence interval of the scores.

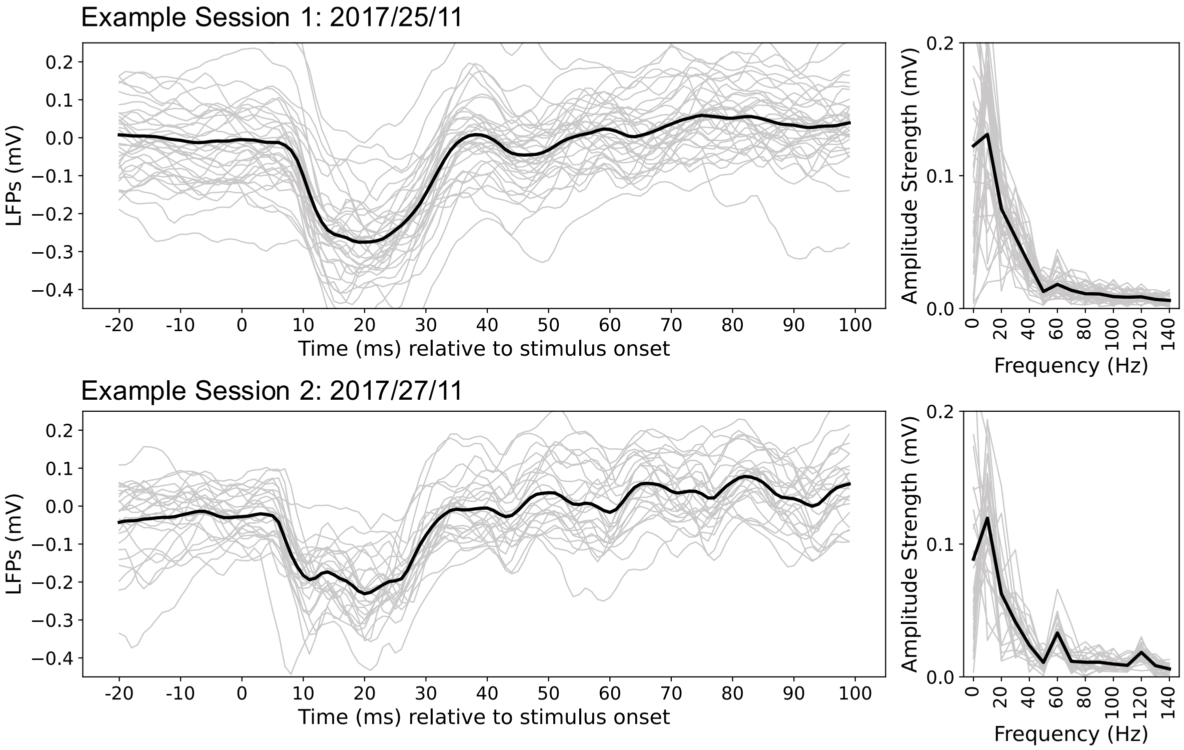

Figure S2 – LFP traces for the evoked response in two example sessions

LFP recordings from two sessions were derived from the L4 electrode for 100% stimulus intensity trials only. Corresponding FFT spectra are shown in the panels on the right-hand side. In the second example, one can clearly see stronger amplitudes for the 60 and 120 Hz components, while this oscillatory activity is not captured in the first example.

Section 1 – Distribution of incorrectly predicted trials

We further investigated the difference between SD and RP models by analyzing how incorrectly predicted trials were distributed over the course of the session. This analysis was motivated by our previous study in which we observed a changing decision criterion related to the decreasing response probability from mice towards the end of the session as mice become more saturated and consequently less motivated to receive the water reward (Vandevelde *et al.*, 2022). Thus, the mouse emits more impulsive licks (i.e., spontaneous licks not evoked by stimulus detection) in the beginning and fewer such licks at the end of the session. Nevertheless, the response of mice was consistently linked to the presentation of a stimulus, and we expect that the RP models eventually learn to detect the stimulus without being able to extract more information from the signal relevant to the discrimination of successful response and no-response trials. As expected, the RP model generates more False-Negatives at the beginning of the session as the model considers catch trials as no-response trials while mice attempt to obtain water even in the absence of a whisker stimulus (**Fig. S2**). Toward the end of the session, the RP model positively detects stimulus trials, which are often response trials as well. However, even for strong stimuli, mice became less responsive as the session progressed, resulting in False-Positive predictions of the model. From these observations, we conclude that the RP model supposedly predicts the response based on stimulus detection without being able to extract statistical patterns relevant to predicting the behavioral response of mice. The scenario investigated here is the one based on the RAW features from layer L4, keeping 0% and 100% intensity trials only.

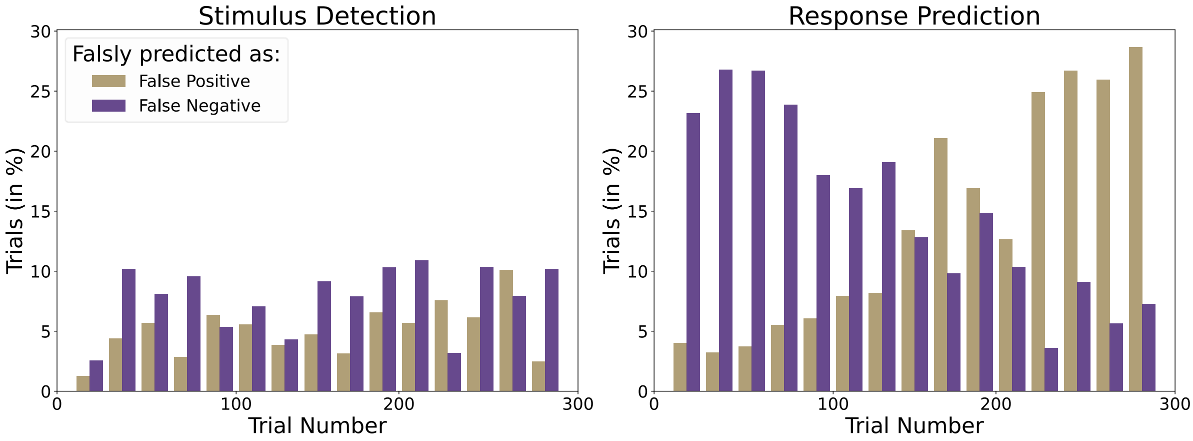

Figure S3 – Distribution of falsely predicted trials

Bars represent the proportion of False-Positive and False-Negative predictions from two types of models: the Stimulus Detection model (left) and the Response Prediction model (right). Trials were binned over the course of the session with a bin width of 20, resulting in 15 bins for 300 trials per session.
